# Food-Induced Anaphylaxis: Role of Hidden Allergens and Cofactors

**DOI:** 10.3389/fimmu.2019.00673

**Published:** 2019-04-03

**Authors:** Isabel J. Skypala

**Affiliations:** Department of Allergy and Clinical Immunology, Imperial College, Royal Brompton and Harefield NHS Foundation Trust, London, United Kingdom

**Keywords:** anaphylaxis, food, allergy, hidden, allergens, co-factors

## Abstract

Food anaphylaxis is on the increase, with those who have an allergy to peanuts, tree nuts, milk, and seafood at the highest risk of developing such a reaction. However, the diet in many societies is increasingly varied, much of the food consumed is prepared outside the home, and meals are often composed of many different ingredients. Anaphylaxis may occur to a composite food, and it may be unclear whether the reaction is due to contamination or to a culprit allergen present in an added ingredient. Composite foods can contain many allergic proteins present in small amounts, which do not always have to be labeled, unless they feature in European or US labeling regulations. These “hidden” allergens include mustard, celery, spices, lupine, pea, natural food colourings, and preservatives, but can occasionally include allergenic material from contaminants such as cereal mites. Hidden allergens can provoke severe reactions to seemingly unconnected foods which might then lead to a diagnosis of idiopathic anaphylaxis. The same problem can arise with two well-known types of food allergy; wheat-dependant exercise induced anaphylaxis and allergy to non-specific Lipid Transfer Protein allergens, both of which might only manifest when linked to a cofactor such as exercise. Many of these risk factors for food anaphylaxis have a common link; the public's engagement with popular concepts of health and fitness. This includes the development of a food and exercise culture involving the promotion and marketing of foods for their health-giving properties i.e., meat substitutes, wheat substitutes, supplements and alternative, or “natural” remedies for common ailments. Some of these foods have been reported as the cause of severe allergic reactions, but because they are often viewed as benign unlikely causes of severe allergic reactions, could be considered to be hidden allergens. The best resource to elicit the likelihood of a hidden allergen provoking an allergic reaction is to take a detailed history of the allergic reaction, presence of co-factors, foods suspected, type of food and where it was consumed. A good knowledge of commonly used ingredients, and list of potential hidden allergen suspects are essential tools for the food allergy detective.

## Introduction

Many foods have the potential to provoke an allergic reaction, depending on the individual susceptibility, the threshold dose required to elicit an allergic reaction and the type of allergens present within the food. However, a study by Wainstein et al. showed clearly that the amount of allergen which provokes a reaction in a food challenge does not predict the severity of the reaction or the likelihood of anaphylaxis (1). Turner et al. surmised that anaphylaxis outcome and severity cannot be determined by the eliciting dose, (amount/kilogram body weight) and that sensitivity and severity need to be considered separately by distinguishing between the amount and “dose,” which will differ significantly between young children and adults (2). It is clear some foods are more likely to provoke anaphylaxis from the numerous publications reporting anaphylaxis data in large cohorts of patients. A systematic review of fatal anaphylaxis found that peanut and tree nuts are the most common triggers, implicated in up to 87% of deaths, although other foods including milk and seafood were also important causes of mortality in some countries (3). A review by Parrish and Kim reported that an allergy to milk, seafood, peanuts or tree nuts increased the risk of fatal anaphylaxis, whereas allergy to egg and soy carried a decreased risk (4). The European anaphylaxis registry reviewed 1970 cases of anaphylaxis in children up to the age of 17 years. Food was a trigger in 66% of reactions with milk and egg being important elicitors in very young children and tree nuts in older children, but although peanuts were the most important elicitor of reactions overall, affecting 28% of the children, 11% had reactions to fruits and vegetables, 4% to spices, and 5% to legumes other than peanut or soy (5). So although peanut and tree nut are implicated in anaphylactic reactions, other foods are also important causes. The type of foods involved may vary with age, but also with geography. Jiang et al reviewed 1952 episodes of anaphylaxis in 907 Chinese patients across a wide age range, 77% of which were attributed to a food (6). Wheat was the most common trigger overall with some variation depending on the age range, but fruits and vegetables accounted for 20% of anaphylactic reactions and spices were the trigger in 25 reactions.

A 10-year survey of cases of food anaphylaxis reported to the food allergy register in Norway found that the commencement of using lupin flour in bakeries led to increased number of peanut allergic patients reporting reactions to lupine, and also to fenugreek, a legume commonly used in spice mixes (7). The authors reported these as examples of “hidden” allergens in composite foods, and linked their occurrence to their relatively recent introduction into the Norwegian diet. Nanagas et al. undertook a wide-ranging review of hidden allergens, which included non-foods including such as aluminum and also some so-called exotic foods such as crocodile, sago worms (8). New foods and food trends mean that many meals eaten are composite meals, the composition of which might be difficult to elicit. Trends for food consumption suggest an increase in the use of ready-made products. Moubarac et al. reported that the most notable change in Canadian dietary habits over a 70-year period was the increase in the use of ready to eat meals, rising from 28% in 1938 to 61% in 2011 in the Canadian population (9). Thus, there has been a displacement of unprocessed foods by ready to eat meals which can contain a variety of additives and food allergens. This has been paralleled by an increased online promotion of nutritional and lifestyle advice, which can advocate the mass consumption of concentrated forms of fruits, vegetables, soy products and nutritional supplements. Online information is persuasive; Ross et al found that over 85% study participants followed advice on food allergy obtained through the internet or social media (10).

## Methods

Jappe et al. described hidden allergens as one which are either deliberately added to food but not listed in the ingredients or an allergen present unintentionally due to cross-contamination (11). However, a hidden allergen can also be one which is added deliberately and labeled but is a very minor part of a composite dish and therefore either overlooked or not considered a likely cause due to the norms of food allergy in that particular country. The selection of foods for mandatory labeling varies depending on the priority given to specific foods in certain countries. Some hidden allergens such as mustard and celery appear on some lists but not on others (11). Some of the hidden allergens are common ingredients in seasoning mixes or added due to their particular properties in determining the final quality of the food. They might be ones which people can react to at very low levels. Sharma et al. suggested that hidden allergens should include honey, cinnamon, lupin, pea, mustard, paprika, ginger, oregano, and garlic (12). Food processing and the food matrix also have an effect on the allergic potential of a food, due to its impact on the stability and structure of proteins which is particularly important given the increase in ready prepared meal consumption (13). A literature search was undertaken using the keywords, but also by allergy to individual foods and food additives. The results demonstrated the very wide range of foods involved in hidden anaphylaxis and it was not possible to include them all. So those which might be less obvious were chosen, and grouped into (i) foods used as ingredients, (ii) food additives, (iii) foods eaten for health reasons, (iv) co-factors, and (v) other sources of hidden allergens.

## Results

### Foods Used as Ingredients

The strong flavor of celery means it is often added to many ready-prepared foods. Celery has long been recognized as a problem food allergen (14). One reason for this might be the increasing levels of pollen sensitization. People with pollen-related food allergy, known as Pollen Food Syndrome (PFS) have a greater risk of reactions to celery due to the cross reactivity between the major allergen in Birch pollen (Bet v 1) and the homologous PR10 allergen in celery (Api g 1) (15). Celery can also provoke symptoms in those sensitized to Artemisia pollen, due to cross-reactions between the profilin allergens Api g 5 and Art v 4, hence the term celery-birch-mugwort-spices syndrome which can involve a huge range of foods including celery, mustard and other spices (15). It has been traditionally considered that PFS only occurs to raw plant foods, due to the PR-10 allergens involved losing their allergenic activity after heating causes protein unfolding (16). Lyons et al. found that 46% of celery allergic individuals could tolerate cooked celery probably due to the unfolding of Api g 1 during heating (17). However, Sancho et al. reported that the IgE binding activity of Api g 1 remained stable during pepsin digestion, when compared with other PR10 allergens in apple, peach and hazelnut (18). The resistance to both heat and digestion of the PR10 allergen might explain why Ballmer-Weber et al. reported that over 50% of patients allergic to celery had a positive blinded oral food challenge to cooked celery, and celery spice (19). Rib-Schmidt et al. determined that Api g 1 is susceptible to pH and thus the type of processing may influence the likelihood of reactions in celery allergic individuals (20). It has also been demonstrated that people who have celeriac allergy have symptoms at very low doses, with the estimated dose likely to elicit reactions in 10% of a study population (ED10) for celeriac was 1.6 mg protein, compared to the 2.8 mg of peanut protein and 8.5 mg of hazelnut protein (21). In addition to provoking food allergic symptoms in the traditional way, celery is one of the foods linked to Food-Dependent, Exercise-Induced Anaphylaxis (FDEIA), a condition whereby reactions to foods occur in conjunction with exercise and other co-factors (22, 23). One allergen family closely associated with co-factor induced reactions are the non-specific Lipid Transfer Proteins (LTP), plant food allergens stable to heat and digestion and capable of triggering severe allergic reactions, especially linked to exercise or the consumption of alcohol and non-steroidal anti-inflammatory drugs (NSAID) (24). One LTP identified in celery, Api g 2, has been shown to be a relevant allergen in the celery stalk and thus might be an important cause of allergic reactions to celery in populations where the celery stalk is more commonly eaten, or where LTP allergy is manifest (25).

Another food commonly used for its strong flavor properties is mustard. All mustard plants are members of the Brassicaceae family, and include Brassica juncea (brown/Indian mustard) and Brassica nigra (black mustard), both of which are more pungent than Sinapis alba (yellow mustard), and thus the color and strength of mustard used in a particular region will depend on the mix of mustard seed varieties used (12). As well as being a popular condiment, mustard is often an added ingredient in composite foods such as pizza, savory tarts and ready meals, so may often be overlooked as an allergen (26). Mustard has been shown to be very allergenic, with an eliciting dose of 0.05 mg, one of the lowest eliciting doses of the main allergenic foods (27). The prevalence of allergy to mustard is variable and will depend on the extent of consumption; for example, in France it has been shown to be a common food allergen in children (28). Mustard allergy has also been significantly associated with reactions to nuts, legumes, corn and Rosaceae fruits, which can be explained by the variety of different allergens in mustard able to cross-react with other foods (29). The most prevalent allergen in Sinapis alba is Sin a 1, a 2S Albumin (30). Another heat stable allergen, the 11S Globulin Sin a 2, has shown cross-reactivity to tree nuts and peanuts, and is also associated with severe reactions in mustard allergic patients, possibly due to its interaction with lipid components which may make it resistant to gastro-intestinal degradation (31, 32). Vereda et al. demonstrated that 25/32 patients tested had a positive skin prick test to Sin a 1 and 19/32 to Sin a 2; the majority of whom had experienced immediate systemic reactions after the ingestion of mustard (30). Two other allergens, Sin a 3, an nsLTP, and Sin a 4, a profilin, are present in yellow mustard seed, but in much smaller amounts, and are less likely to be positive in mustard allergic individuals (30, 33). Patients who have a mustard allergy with sensitization to Sin a 3 are more likely to have an allergy to other LTP foods such as peaches (30, 34) Sin a 4 is less likely to be involved in reactions to hidden allergens, as typically reactions are due to cross-reactivity to mugwort pollen (35).

In addition to celery and mustard, herbs and spices have sometimes been involved as hidden allergens. Some herbs have also been linked to anaphylaxis including coriander used as a “natural” flavoring in beer; flavorings in alcoholic drinks do not have to be individually declared on the label (36). Cumin rarely causes reactions to foods, although isolated reports of anaphylaxis to cumin exist, but due to allergen similarity it can be mistaken for a much more allergenic food; undeclared peanut and almond in a taco spice was subsequently found to be cumin (37). Data from the French CICBAA database suggests that only adults are sensitized to spices, but sensitization and allergy to spices such as paprika, cayenne pepper and peppercorns is rare (38). Black, green and white pepper are all derived from Piper nigrum, but pink peppercorns are a dried berry of the shrub *Schinus molle* from the *Anacardiaceae* family; cashew nut is also a member of the same botanical family. A study on children with suspected cashew nut found that of the 36 sensitized to cashew nut, 19% were co-sensitized to *Anacardiaceae* species, and albumin- and legumin-type seed storage proteins sequenced in pink peppercorns cross-reacted with the serum of the cashew nut-sensitized individuals (39). Sumac is another spice belonging to the *Anacardiaceae* family. It is rarely cited in the literature as an allergenic food but Che et al reported a case of a 14-year-old girl with a known cashew nut allergy, who developed symptoms after eating a Fattoush salad containing Za'atar, a mixture of spices consisting of sumac, sesame, thyme, and salt. Subsequent skin prick tests to sumac were strongly positive (40).

One spice which can be very troublesome is fenugreek (*Trigonella foenum-graecum*), a member of the Fabaceae or Legume family. Fenugreek leaves are used as a herb and the seeds as a spice, so fenugreek is often a constituent of spice mixes such as curry powder. It is also considered that fenugreek has a number of health benefits due to its antioxidant, and anti-inflammatory effects, so it is often used in traditional medicine (41). It has been suggested that reactions to fenugreek are usually linked with a primary allergy to peanut. The Food Allergy register in Norway reported that reactions to spicy foods and Indian dishes by peanut allergic individuals were discovered to be due to fenugreek (42). In a case report, a 14-year-old boy with a peanut allergy had a reaction after eating a curry known to be peanut free, and was found to be strongly sensitized to fenugreek (40). The cross-reaction between these two legumes might be due to the homology between fenugreek allergens and the peanut allergens Ara h 1 and Ara h 3 (43). Other case reports include reactions to fenugreek in both children and adults without any known food allergies, so it is important to take a really good history of foods eaten and include fenugreek in any testing where a composite meal has been eaten (44–46).

Other legumes can be causative of hidden allergen reactions, especially those not required to be labeled on food products such as pea, lentil and chickpea. All of these legumes are utilized in different countries in the manufacture of a wide range of foods. One legume which does have to be labeled in European countries is lupin, which has become more utilized in many countries as a replacement to soy (47). So, like mustard or celery, lupin might be present in small amounts, and therefore can act as a hidden allergen. Lupin flour might be added to wheat flour or be present in gluten-free pasta, the cause of the reaction in the first case report to be published on lupin allergy, with subsequent reports involving reactions to a diverse range of foods including cookie, a chocolate and a particular brand of hot dog bread (48–51). A study of a large cohort of 1160 subjects reported that 4.1% were sensitized to lupin, and there was a 75% co-sensitization rate between lupin and legumes (52). In contrast, a much lower level of sensitization was seen in a Norwegian study, with 1.6% (25 subjects) of 1,522 patients with suspected food allergy sensitized to lupin, seven of whom had a positive oral food challenge (53) Peeters et al. characterized 39 peanut sensitized individuals, reporting that 82% were sensitized to lupin, and 35% of the study population had a positive oral food challenge to lupin, with the lowest eliciting dose (ED) being 0.5 mg (54). In a review in 2016, Mennini et al. reported that the prevalence of lupin allergy is still unknown and will vary depending on the dietary norms of the country, but they suggest that sensitization to lupin will occur in 15–20% of individuals with a peanut allergy (55). The major lupin allergen in *Lupinus angustifolius*, is Lup an/a β-conglutin (7S globulin), which has also been demonstrated to be the major lupin allergen in a population of peanut-allergic Italian children (56, 57). There is cross-reactivity between β-conglutin and the peanut allergen Ara h 1, and also between δ-conglutin and Ara h 2, the 2S Albumin in peanuts (58). Other allergens identified include a profilin, a PR10 protein and an LTP allergen (47). Unlike some other legumes, various heating methods including boiling, cooking and microwaving does not affect the stability of most lupin allergens (59).

As well as being major or minor components of composite foods, some legumes are also used as additives. Guar beans (*Cyamopsis tetragonoloba)* are also members of the Fabaceae family, and the polysaccharide extracted from them, guar gum, has thickening and stabilizing properties which makes it an important additive in foods such as baked foods, thickened milk products, condiments, and canned soups. Reactions to guar gum are most often linked to occupational allergy although there has been one case report of an anaphylactic reaction to guar gum (60, 61). The same paper also reported on the allergic potential of other gums including carob gum, tragacanth gum, acacia gum, and pectin, with case reports demonstrating that allergic manifestations can occur across the whole spectrum of symptom severity, and also that reactions may be more likely to occur through occupational exposure. Pectin is often found in sweets, jellies and jams, and reactions to pectin seem to be more likely to occur in those who are sensitized or allergic to cashew nuts (62). Gelatine has also been reported as a rare cause of food anaphylaxis, with a link in some patients to sensitization to galactose-α-1,3-galactose (alpha-gal) (63).

### Food Additives

Some food additives can provoke reactions in small amounts, and unlike foods, most do not fall under mandatory labeling law. Artificial food colorings were the focus of many studies in the latter part of the twentieth century, with tartrazine and sunset yellow being prime examples of additives of concern. The public reaction against artificial colors led to the resurgence of natural food colorings, which conversely can provoke reactions due to the retention of allergenic protein. One such coloring is carmine or cochineal, a red pigment extracted from female parasitic insects (*Dactylopius coccus*). Anaphylaxis to cochineal had been reported by Wüthrich et al. in the late 1990s which led to both the European Union and USA Food and Drug Administration mandating new rules regarding the labeling of cochineal in foods and cosmetic products (64). In 2018, Takeo et al. undertook a review of 22 cases of cochineal allergy in Japanese women, and reported that foods, drinks and cosmetics triggered reactions (65). Other colorings both natural and artificial have been linked to reported allergic reactions, but these are very rare events (60). Sodium metabisulphite is one of the main preservatives which might be responsible for unexplained reactions to composite foods. The use of sulphites is carefully controlled in both Europe and the USA, due to a number of severe reported reactions including anaphylaxis in the latter part of the twentieth century (66). Both the European Union and the US Food and Drug Administration require foods containing more than 10 ppm (10 mg/kg) to be labeled, but there is significant variation between levels for the same foods (67). The likelihood of reactions to sulphite are greater in those who have asthma; Vally and Misso suggested that 3–10% of asthmatics experience symptoms on exposure to ingested sulphites (68). Foods likely to contain higher levels of sulphite include, white and rose wine, light colored fruit juices, dried fruits and vegetables and salt cod (69).

### Foods Eaten for Health Reasons

A survey of Australian adults reported that 7·3 % of the population were avoiding wheat, and similar data was obtained from a survey of 785 adults in the Netherlands, which found 6.25% had stopped eating wheat due to concerns relating to gluten intolerance (70, 71). Online promotion of gluten-free (GF) diets has undoubtedly influenced the increase in the avoidance of wheat, but wheat substitutes have all been linked to cases of allergy including anaphylaxis to oats (72). Boussalt et al. reported that 32% of those who regularly used oat-based creams had oat-positive atopy patch test (APT) whereas none of the non-user were sensitized (73). Other foods consumed more often if wheat is being avoided include quinoa, buckwheat, millet, and amaranth. Hemmer et al. identified nine adult patients who reacted on first oral exposure to millet with moderate to severe systemic reactions including anaphylaxis; 8/9 reported to have kept budgerigars either as adults or children which were regularly fed with millet (74). In a review of 991 cases of anaphylaxis in Korean children and adults, buckwheat was identified as the causative allergen in 6.5% of food allergy cases, and in another study 17/ 26 buckwheat allergic children (65.4%) had anaphylaxis (75, 76). Although buckwheat allergy is not as frequent in Europe, it is important to consider it as a potential cause of hidden anaphylaxis (77, 78). Amaranth seed is a staple food in some countries but in others it is used as a “grain” substitute for wheat, and has also been reported to cause anaphylaxis (79). Like amaranth and buckwheat, quinoa is not a cereal, but functions as a very popular cereal substitute, first reported as an anaphylaxis trigger in 2005 by Astier et al. (80). Another case from the United States of America concerned a 29-year-old woman who developed allergic symptoms including a sensation of choking, 5 min after eating a quinoa salad (81). Gluten-free products in many countries also often contain lupin. Patients with coeliac disease also have the possibility to also develop wheat allergy. Micozzi et al. presented two cases of children with coeliac disease who subsequently had allergic reactions to wheat due, it is proposed, by sensitization to the trace amounts of wheat in gluten-free foods (82). Another group from Spain also published case reports of two children with coeliac disease who developed allergic reactions immediately after accidental exposure to wheat, with the wheat allergen Tri a 14 (wheat LTP) the responsible allergen (83). There are few documented case reports of milk substitutes provoking anaphylaxis, but Gly m 4, the allergen in soy milk which cross-reacts to the main birch pollen allergen Bet v 1, has been shown to be capable of provoking severe reactions (84).

Meat substitutes used to be mainly soy based products, but in the last 20 years a fungus called *Fusarium venenatum* has been used as the basis for a popular meat substitute. This mycoprotein has also been linked to a large number of reported allergic reactions, many involving anaphylaxis. Jacobson et al. reported that 312 of the reported reactions to mycoprotein were indicative of an allergic reaction, with anaphylaxis reported in 72% of cases (85). Composite dishes containing mycoprotein may not always be immediately obvious as the mycoprotein takes on the flavor of the herbs and spices used in the dish, and thus it might be mistaken for minced meat or chicken. Two case reports from 2002 and 2003 also highlighted a possible link between sensitization to mold and allergic reactions on the ingestion of mycoprotein (86, 87).

There has been an increase in the number of people taking large quantities of fruits and vegetables in the form of juices or smoothies, linking in with the rise of so-called superfoods. A superfood is a nutrient-rich food considered to be especially beneficial for health and well-being. Commonly cited superfoods include blueberries, goji berries, pomegranate juice, broccoli, garlic, and beetroot. The public perception is that superfoods are universally good for you and thus less likely to cause an allergic reaction, although a large retrospective study on 2.7 million patients in the USA determined that the prevalence of allergy to fruits and vegetables was 0.7% (88). The most prevalent allergy involving fruits and vegetables is PFS, which generally manifests with mild to moderate oro-pharyngeal symptoms and therefore is not usually linked to severe or anaphylactic reactions (89). However, concentrated forms of fruits and vegetables, such as fresh fruit smoothies, juices or soy milk can cause more severe reactions in those with PFS (84). Lipid transfer proteins are heat stable allergens which are highly cross-reactive and unlike the allergens involved in PFS, they occur in both raw and cooked foods. Gogi berries and pomegranate are two foods which have the potential to provoke severe reactions due to their LTP allergens (90, 91).

Increasingly food products are being used in complementary medicine. In a recent review Gunawardana outlined the causes of anaphylaxis linked to complementary medicine products, reporting that the main products to elicit anaphylaxis included the use of *Andrographis paniculata*, Echinacea species (dandelion, ragweed, mugwort, golden lion and sunflower), bee products, *Ginkgo biloba*, and Ginseng (92) The other main product which can provoke allergic reactions is psyllium or ispaghula, a natural hydrophilic mucilloid of the genus Plantago, traditionally used as a laxative and also known to have lipid-lowering effects. Khalili et al. documented a case of death from anaphylaxis due to psyllium and concluded that it should no longer be considered an emerging food allergen; it is now firmly established as a potential life-threatening allergen (93).

### Co-factor Induced Reactions

Co-factor induced anaphylaxis is also another good example of anaphylaxis where the trigger might be considered to be a hidden food. This is because co-factor induced food allergy only occurs when a food is eaten in proximity to a co-factor such as exercise, alcohol, or non-steroidal anti-inflammatory drugs (NSAID). The first co-factor linked to reactions to foods was exercise, with FDEIA occurring when food is consumed in close proximity to exercise, and wheat being the food most closely associated with this condition (94). Much of the data for FDEIA focusses on wheat triggers. Christensen et al. investigated 71 patients with suspected Wheat-Dependent Exercise Induced Anaphylaxis (WDEIA), reporting that two thirds had a positive wheat and exercise challenge, one third of whom reacted to wheat alone although the median dose required was double that required to elicit a reaction to wheat and exercise combined (95). They concluded that exercise lowers the threshold and increases the severity of the reaction to the food. This concept therefore means that people diagnosed with FDEIA need to be aware that eating large amounts of a food when not in conjunction with a co-factor could increase their risk of having anaphylaxis. The same might be true for other foods, especially in people with a diagnosed LTP allergy, who appear to be more likely to have a reaction to the food when a co-factor is present (96, 97). Cardona et al. evaluated 74 cases of suspected co-factor enhanced food allergy and reported that NSAID were involved in 58%, exercise in 52.7%, and alcohol in 12.2%, with LTP allergens being the allergen most frequently involved (98). One of the most recent publications suggests that co-factors may include more than exercise, NSAID or alcohol. Versluis et al. found that 74% of 157 patients reported cofactors to be present during their allergic reactions, most often tiredness (38%), alcohol intake (16%), symptoms of pollinosis (16%), and stress (99). A review in 2018, referenced many other co-factors including menstruation, infection, extreme air temperatures, cannabis use, and medications other than NSAID, including angiotensin-converting enzyme inhibitors, beta-blockers and antacids (100). It has been suggested that the frequent involvement of exercise and NSAID in food-induced anaphylaxis highlights the importance of their recognition and inclusion into a diagnostic workup (101).

### Other Sources of Hidden Allergens

Hidden allergens can include those which are present due to contamination or infestation, rather than being a composite part of the food. Sánchez-Borges et al. published a list of foods most likely to contain cereal mites, which most commonly included pancakes, other flour containing foods but also cheese, ham, chorizo, and salami (102). The common culprit is D. farinae, but other species been implicated as inducing oral mite anaphylaxis which is more likely to be a problem in geographical locations with high temperatures (>278C) and humidity (>70%) (103). Cases are not always obvious; a 71-year-old man with no history of food allergy or atopy suffered anaphylaxis after a meal of grits and shrimp, found to have been provoked by D farinae which had infested the grits, which had been in the store cupboard for a year (104). Another recent case report involved three Irish medical students diagnosed with oral mite anaphylaxis after eating home-cooked pancakes (105). Another type of contamination is also due to infestation by Anisakis simplex, a parasite which can infect fish such as cod and salmon (106). The Netherlands was the first country to report on the existence of Anisakiasis, but the vast majority of reported cases (90%) are from Japan (107). One Italian study reported that 15% of patient screened were sensitized to Anisakis simplex, confirming that eating raw or marinated fish promotes sensitization, thus the likelihood of developing an allergy to Anisakis depends on local eating habits (108). In a review Nieuwenhuizen and Lopata reported that the number of cases of anisakiasis in the Netherlands was reduced to practically zero, following legislation which required all fish meant for consumption to either be deep frozen for at least 24 h or cooked at 60°C for at least 10 min or more (109).

In addition to being present in foods accidentally, insects may be consumed as a food in some communities, either as a traditional constituent of the diet, or due to increasing awareness of food sustainability. Steffie de Giera and Kitty Verhoeckxa undertook a major review of insects as foods and allergen sources in the diet (110). They included information from 30 case reports centered on a variety of different insects including beetles, silkworms, caterpillars, locusts, grasshoppers, bees, and *Dactylopius coccus*, the insect involved in cochineal allergy. They concluded that allergens such as tropomyosin, arginine kinase, and others, which are commonly involved in allergy to crustaceans, mollusks, house dust mite and cockroach, may also be important allergens in reactions to insects especially in those who are sensitized to house dust mite, shrimp, prawn, and crab.

Food allergens can also be present in non-food items. In an extensive review of hidden allergens, Baker et al. reported on a number of instances of foods causing reactions when part of a medicinal product, such as milk protein contamination of lactose in an inhaler (62). Tamagawa-Mineoka et al. reported one patients who became allergic to corn and another who reacted to various foods after using a soap containing oats and almonds (111). A child's fingerprint testing kit provoked anaphylaxis in a wheat-allergic 9-year-old boy, was found to contain wheat in the fingerprint powder, which was causing a reaction when blown off the fingerprint paper (112). Buckwheat hulls, also known as sobakawa hulls or sobagara husks used in pillows have been reported to induce asthma and allergic rhinitis (113). Other non-food anaphylaxis may involve sensitization to insects. A case reported by Makatsouri et al. involved an anaphylactic reaction occurring in a peanut allergic individual, 5 min after she tried on a silk blouse and silk dress, who was subsequently shown to be sensitized to silkworms (114).

## Discussion

A study reviewing the causes of “idiopathic” anaphylaxis in UK subjects reported that a food allergic cause was determined through microarray technology in 20% of those so labeled (115). Hidden causes of anaphylaxis are often difficult to determine, as they can be caused by a flavoring, additive, other food component or food supplement—see Table 1. The lupin “epidemic” in Norway in the early part of this century demonstrates how the introduction of novel food proteins into a community brings with it the likelihood of allergic reactions. Thus, the increase in new sources of food protein, such as insects, may inevitably result in further manifestations of food allergy in those already sensitized to common allergenic proteins such as tropomyosin. In young children, there is an easier path to tread in terms of determining food triggers, whereas teenagers, and adults, who are frequently consuming meals and snacks of unknown origin, are not so straightforward. Increasing globalization and social media has fueled a desire by many in the Western world to consume novel foods, health-related shakes, smoothies or juices, or complementary therapies which contain food. In an age where much food eaten is prepared outside the home, the cause of anaphylaxis can be difficult to determine.

**Table 1:** Common hidden allergens and associated foods.

**Hidden allergen**	**Foods most likely to contain the hidden allergen**	**Potential cross-reacting allergens:**
Celery	Soups, meat dishes, sauces, gravy, pizza, ready meals, seasoning	Birch, mugwort, mustard, spices
Mustard	Soups, meat dishes, sauces, pizza, ready meals, cheese dishes, curries, barbeque, other spicy dishes. Mustard seeds might also be found in breads and crackers	Nuts, legumes, corn and Rosaceae fruits especially peach
Pink peppercorns	Could be added as a garnish	Cashew nut
Sumac	Moroccan, Mediterranean, Israeli food and dishes from the Middle East. Za'atar is a condiment which contains sumac, hyssop, sesame and spices	Cashew nut
Fenugreek	The leaves, seeds and roots are used in many cuisines including Indian, Turkish, Iranian and Egyptian dishes. Spice mixes, curry powder, panch phoron, and sambar powder may all contain fenugreek. Also used as a herbal remedy	Peanut
Lupin	Beans eaten as a snack, especially in Mediterranean countries and South America. Lupin flour may be present in pastry products, pancakes, pizza, batter and gluten-free products	Peanut
Pectin	Mainly in jams and jellies, but can also be present in desserts, confectionary and medicines	Cashew nut
Cochineal or Carmine	Sweets, ice lollies, meat products, soft drinks, colored cakes and biscuits, syrups, lipstick and other makeup	House dust mite
Sodium metabisulphite	Wine, beer, cider, dried fruits, sausages, burgers, prawns, mycoprotein	
Buckwheat	Galettes (French savory pancakes), noodles, gluten-free products, buckwheat pillows, soap	
Oats/Oatmeal	Breakfast cereals, biscuits, soap and bath products, gluten-free products	
Mycoprotein	Vegetarian/Vegan ready meals	Molds
Psyllium or Ispaghula	Laxatives. Also used as a thickener in ice cream and desserts	
Cereal mites	Stored flour or cereal products especially pancakes and other foods made from wheat flour or cornmeal such as pizza, breadsticks, breadcrumbs	House dust mite
Anisakis	Raw or marinated fish	

The allergy history has been shown to be extremely important in determining food triggers and patterns, so the most important part of an allergy history where anaphylaxis has been reported is in determining which foods are tolerated by the patient (116). Understanding the dietary sources of hidden allergens can be a challenge, especially where the population is diverse and may consume traditional foods, the composition of which is not always easy to determine. Unusual triggers of anaphylaxis may occur in specific populations where traditional foods or those indigenous to the region are consumed. In Jiang's study of Chinese anaphylaxis, chrysanthemum tea, bullfrogs, locusts, cicadas and silkworm chrysalis were all reported causes of anaphylaxis (6). It is therefore important to keep an open mind about unusual causes, if the history elicits a clue as to a potential trigger however unlikely this might be. Taking a patient through the meal or snack eaten in the hours prior to the anaphylactic reaction is vital, bearing in mind that if a food has subsequently been consumed without consequence this might not automatically remove it from the list of suspects if co-factors have been involved. Table 1 shows which hidden allergens are most likely to be present in pre-prepared meals and snacks, but this cannot necessarily translate across countries or continents, and personal knowledge of the typical foods consumed by local populations is therefore vital to the diagnostic process. Pre-existing food allergies to peanut or cashew nut may indicate a greater likelihood of reactions to foods known to cross-react such as fenugreek, lupin, sumac, and pink peppercorns. The presence of sensitization to pollens or LTP allergens may also be helpful in determining the cause of reactions to apparently unconnected foods. Cross-reactions between plant foods and pollens, such as in PFS, or due to highly homologous proteins in different plants, such a latex-food cross-reactions or LTP allergy, are well recognized (117). However, other cross-reactions are less well documented and can be the cause of hidden anaphylaxis, such cross-reactivity between beef and milk, for which a positive bovine serum albumin is a diagnostic marker (118). This allergy has been shown to be the cause of immediate hypersensitivity reactions to vaccines containing bovine/porcine excipients (119). The other cross-reactive food allergy involving meat is due to sensitization to the galactose-α-1,3-galactose (alpha-gal) through tick bites, which can cause anaphylaxis, angioedema, or urticaria to mammalian meats 3–6 h after ingestion (120). This phenomenon may well manifest as idiopathic anaphylaxis, and although first and most often reported in the USA, has also been observed in Central and South America, Europe, Asia, Scandinavia, and Australia (121).

Our understanding of the causes of hidden anaphylaxis is greatly helped not only by large studies, but also individual case reports which demonstrate the importance of keeping an open mind, taking a very detailed history of the foods consumed, and improving our knowledge of food ingredients and likely contamination issues. In a review article, Jappe and Vieths suggest the importance of re-evaluating a diagnosis of idiopathic anaphylaxis in case a hidden allergen, such as lupin, is present (122). The goal for our patients must be a definitive diagnosis where possible, rather than a label of idiopathic anaphylaxis, so they can safely enjoy eating and avoid the risk of social isolation.

## Author Contributions

The author confirms being the sole contributor of this work and has approved it for publication.

### Conflict of Interest Statement

The author declares that the research was conducted in the absence of any commercial or financial relationships that could be construed as a potential conflict of interest.

## References

[1] Wainstein BK Studdert J Ziegler M Ziegler JB. Prediction of anaphylaxis during peanut food challenge: usefulness of the peanut skin prick test (SPT) and specific IgE level. Pediatr Allergy Immunol. (2010) 21:603–11. 10.1111/j.1399-3038.2010.01063.x 20444154

[2] Turner PJ Baumert JL Beyer K Boyle RJ Chan CH Clark AT. Can we identify patients at risk of life-threatening allergic reactions to food? Allergy. (2016) 71:1241–55. 10.1111/all.12924 27138061

[3] Pouessel G Turner PJ Worm M Cardona V Deschildre A Beaudouin E. Food-induced fatal anaphylaxis: from epidemiological data to general prevention strategies. Clin Exp Allergy. (2018) 48:1584–93. 10.1111/cea.13287 30288817

[4] Parrish CP Kim H. Food-Induced anaphylaxis: an update. Curr Allergy Asthma Rep. (2018) 18:41. 10.1007/s11882-018-0795-5 29904820

[5] Grabenhenrich LB Dölle S Moneret-Vautrin A Köhli A Lange L Spindler T. Anaphylaxis in children and adolescents: the European Anaphylaxis Registry. J Allergy Clin Immunol. (2016) 137:1128–37. 10.1016/j.jaci.2015.11.015 26806049

[6] Jiang N Yin J Wen L Li H. Characteristics of anaphylaxis in 907 Chinese patients referred to a tertiary allergy center: a retrospective study of 1,952 episodes. Allergy Asthma Immunol Res. (2016) 8:353–61. 10.4168/aair.2016.8.4.353 27126729 PMC4853513

[7] Namork E Fæste CK Stensby BA Egaas E Løvik M. Severe allergic reactions to food in Norway: a ten year survey of cases reported to the food allergy register. Int J Environ Res Public Health. (2011) 8:3144–55. 10.3390/ijerph8083144 21909296 PMC3166732

[8] Nanagas VC Baldwin JL Karamched KR. Hidden Causes of Anaphylaxis. Curr Allergy Asthma Rep. (2017) 17:44. 10.1007/s11882-017-0713-2 28577270

[9] Moubarac JC Batal M Martins AP Claro R Levy RB Cannon G. Processed and ultra-processed food products: consumption trends in Canada from 1938 to 2011. Can J Diet Pract Res. (2014) 75:15–21. 10.3148/75.1.2014.15 24606955

[10] Ross J Fishman J Wang J. Internet and food allergy. What patients are seeking and what they do with the information. J Allergy Clin Immunol Pract. (2017) 5:494–95. 10.1016/j.jaip.2016.06.006 27372598

[11] Taylor SL Baumert JL. Worldwide food allergy labeling and detection of allergens in processed foods. Chem Immunol Allergy. (2015) 101:227–34. 10.1159/000373910 26022883

[12] Sharma A Verma AK Gupta RK Neelabh Dwivedi PD. A comprehensive review on mustard-induced allergy and implications for human health. Clin Rev Allergy Immunol. (2017). 10.1007/s12016-017-8651-2. [Epub ahead of print]. 29159565

[13] Pekar J Ret D Untersmayr E. Stability of allergens. Mol Immunol. (2018) 100:14–20. 10.1016/j.molimm.2018.03.017 29606336 PMC6020993

[14] André F André C Colin L Cacaraci F Cavagna S. Role of new allergens and of allergens consumption in the increased incidence of food sensitizations in France. Toxicology. (1994) 93:77–83. 10.1016/0300-483X(94)90198-8 7974507

[15] Popescu FD. Cross-reactivity between aeroallergens and food allergens. World J Methodol. (2015) 5:31–50. 10.5662/wjm.v5.i2.31 26140270 PMC4482820

[16] Bohle B Zwölfer B Heratizadeh A Jahn-Schmid B Antonia YD Alter M. Cooking birch pollen-related food: divergent consequences for IgE- and T cell-mediated reactivity *in vitro* and *in vivo*. J Allergy Clin Immunol. (2006) 118:242–49. 10.1016/j.jaci.2006.03.011 16815162

[17] Lyons SA Dijk AMV Knulst AC Alquati E Le TM Os-Medendorp HV. Dietary interventions in pollen-related food allergy. Nutrients. (2018) 10:E1520. 10.3390/nu10101520 30332840 PMC6213550

[18] Sancho AI Wangorsch A Jensen BM Watson A Alexeev Y Johnson PE. Responsiveness of the major birch allergen Bet v 1 scaffold to the gastric environment: impact on structure and allergenic activity. Mol Nutr Food Res. (2011) 55:1690–99. 10.1002/mnfr.201100025 21770047

[19] Ballmer-Weber BK Hoffmann A Wüthrich B Lüttkopf D Pompei C Wangorsch A. Influence of food processing on the allergenicity of celery: DBPCFC with celery spice and cooked celery in patients with celery allergy. Allergy. (2002) 57:228–35. 10.1034/j.1398-9995.2002.1o3319.x 11906337

[20] Rib-Schmidt C Riedl P Meisinger V Schwaben L Schulenborg T Reuter A pH and heat resistance of the major celery allergen Api g 1. Mol Nutr Food Res. (2018) 25:e1700886 10.1002/mnfr.201700886 29800504

[21] Ballmer-Weber BK Fernandez-Rivas M Beyer K Defernez M Sperrin M Mackie AR. How much is too much? Threshold dose distributions for 5 food allergens. J Allergy Clin Immunol. (2015) 135:964–71. 10.1016/j.jaci.2014.10.047 25589011

[22] Romano A Di Fonso M Giuffreda F Quaratino D Papa G Palmieri. Diagnostic work-up for food-dependent, exercise-induced anaphylaxis. Allergy. (1995) 50:817–24. 10.1111/j.1398-9995.1995.tb05055.x 8607564

[23] Hompes S Dölle S Grünhagen J Grabenhenrich L Worm M. Elicitors and co-factors in food-induced anaphylaxis in adults. Clin Transl Allergy. (2013) 3:38. 10.1186/2045-7022-3-38 24262093 PMC4176490

[24] Salcedo G Sanchez-Monge R Diaz-Perales A Garcia-Casado G Barber D. Plant non-specific lipid transfer proteins as food and pollen allergens. Clin Exp Allergy. (2004) 34:1336–41. 10.1111/j.1365-2222.2004.02018.x 15347364

[25] Gadermaier G Hauser M Egger M Ferrara R Briza P Santos KS. Sensitization prevalence, antibody cross-reactivity and immunogenic peptide profile of Api g 2, the non-specific lipid transfer protein 1 of celery. PLoS ONE. (2011) 6:e24150. 10.1371/journal.pone.0024150 21897872 PMC3163685

[26] Kanny G Fremont S Talhouarne G Nicolas JP Moneret-Vautrin DA. Anaphylaxis to mustard as a masked allergen in chicken dips. Ann Allergy Asthma Immunol. (1995) 75:340–42. 7583850

[27] Taylor SL Baumert JL Kruizinga AG Remington BC Crevel RW Brooke-Taylor S. Establishment of reference doses for residues of allergenic foods: report of the VITAL expert panel. Food Chem Toxicol. (2014) 63:9–17. 10.1016/j.fct.2013.10.032 24184597

[28] Rance F Kanny G Datau G Moneret-Vautrin DA. Food hypersensitivity in children: clinical aspects and distribution of allergens. Pediatr Allergy Immunol. (1999) 10:33–38. 10.1034/j.1399-3038.1999.101008.x 10410915

[29] Figueroa J1 Blanco C Dumpiérrez AG Almeida L Ortega N Castillo R. Mustard allergy confirmed by double-blind placebo-controlled food challenges: clinical features and cross-reactivity with mugwort pollen and plant-derived foods. Allergy. (2005) 60:48–55. 10.1111/j.1398-9995.2005.00644.x 15575930

[30] Vereda A Sirvent S Villalba M Rodríguez R Cuesta-Herranz J Palomares O. Improvement of mustard (*Sinapis alba*) allergy diagnosis and management by linking clinical features and component-resolved approaches. J Allergy Clin Immunol. (2011) 127:1304–7. 10.1016/j.jaci.2011.01.020 21349575

[31] Sirvent S Palomares O Cuesta-Herranz J Villalba M Rodríguez R. Analysis of the structural and immunological stability of 2S albumin, nonspecific lipid transfer protein, and profilin allergens from mustard seeds. J Agric Food Chem. (2012) 60:6011–8. 10.1021/jf300555h 22594937

[32] Angelina A Sirvent S Palladino C Vereda A Cuesta-Herranz J Eiwegger T. The lipid interaction capacity of Sin a 2 and Ara h 1, major mustard and peanut allergens of the cupin superfamily, endorses allergenicity. Allergy. (2016) 71:1284–94. 10.1111/all.12887 26991432

[33] Sirvent S Palomares O Vereda A Villalba M Cuesta-Herranz J Rodríguez R. nsLTP and profilin are allergens in mustard seeds: cloning, sequencing and recombinant production of Sin a 3 and Sin a 4. Clin Exp Allergy. (2009) 39:1929–36. 10.1111/j.1365-2222.2009.03382.x 20085601

[34] Palacín A Cumplido J Figueroa J Ahrazem O Sánchez-Monge R Carrillo T. Cabbage lipid transfer protein Bra o 3 is a major allergen responsible for cross-reactivity between plant foods and pollens. J Allergy Clin Immunol. (2006) 117:1423–29. 10.1016/j.jaci.2006.01.026 16751008

[35] Tripathi DM Kumar S Gupta A Mehta N A Prospective Study of Mustard Allergy in Allergic Rhinitis Cases. Available online at: http://www.bhj.org.in/journal/2001_4302_apr01/org_261.htm (accessed October 24, 2018).

[36] Brussino L Nicola S Giorgis V Rolla G Beer anaphylaxis due to coriander as hidden allergen. BMJ Case Rep. (2018) 8:bcr-2018-225562. 10.1136/bcr-2018-225562 PMC6088294 30093499

[37] Garber EA Parker CH Handy SM Cho CY Panda R Samadpour M. Presence of undeclared food allergens in cumin: the need for multiplex methods. J Agric Food Chem. (2016) 64:1202–11. 10.1021/acs.jafc.5b05497 26769163

[38] Moneret-Vautrin DA Morisset M Lemerdy P Croizier A Kanny G. Food allergy and IgE sensitization caused by spices: CICBAA data (based on 589 cases of food allergy). Allerg Immunol. (2002) 34:135–40. 12078423

[39] Bastiaan-Net S Reitsma M Cordewener JHG van der Valk JPM America TAHP Dubois AEJ. IgE cross-reactivity of cashew nut allergens. Int Arch Allergy Immunol. (2018) 178:1–14. 10.1159/000493100 30368491 PMC6381869

[40] Che CT Douglas L Liem J. Case reports of peanut-fenugreek and cashew-sumac cross-reactivity. J Allergy Clin Immunol Pract. (2017) 5:510–11. 10.1016/j.jaip.2016.12.024 28283164

[41] Ouzir M El Bairi K Amzazi S. Toxicological properties of fenugreek (*Trigonella foenum graecum*). Food Chem Toxicol. (2016) 96:145–54. 10.1016/j.fct.2016.08.003 27498339

[42] Faeste CK Namork E Lindvik H. Allergenicity and antigenicity of fenugreek (*Trigonella foenum-graecum*) proteins in foods. J. Allergy Clin. Immunol. (2009) 123:187–94. 10.1016/j.jaci.2008.09.012 18930518

[43] Faeste CK Christians U Egaas E Jonscher KR. Characterization of potential allergens in fenugreek (*Trigonella foenum-graecum*) using patient sera and MS-based proteomic analysis. J Proteomics. (2010) 73:1321–33. 10.1016/j.jprot.2010.02.011 20219717 PMC2894366

[44] Joseph NI Slavin E Peppers BP Hostoffer RW Jr. Fenugreek anaphylaxis in a pediatric patient. Allergy Rhinol. (2018) 9:2152656718764134. 10.1177/2152656718764134 29977649 PMC6028160

[45] Patil SP Niphadkar PV Bapat MM. Allergy to fenugreek (*Trigonella foenum graecum*). Ann Allergy Asthma Immunol. (1997) 78:297–300. 10.1016/S1081-1206(10)63185-7 9087156

[46] Ohnuma N Yamaguchi E Kawakami Y. Anaphylaxis to curry powder. Allergy. (1998) 53:452–54. 10.1111/j.1398-9995.1998.tb03924.x 9574894

[47] Cabanillas B Jappe U Novak N. Allergy to peanut, soybean, and other legumes: recent advances in allergen characterization, stability to processing and IgE cross-reactivity. Mol Nutr Food Res. (2018) 62:1700446. 10.1002/mnfr.201700446 28944625

[48] Hefle SL Lemanske RF Jr Bush RK. Adverse reaction to lupine-fortified pasta. J Allergy Clin Immunol. (1994) 94:167–72. 10.1053/ai.1994.v94.a54942 8064069

[49] Faeste CK Løvik M Wiker HG Egaas E. A case of peanut cross-allergy to lupine flour in a hot dog bread. Int Arch Allergy Immunol. (2004) 135:36–9. 10.1159/000080041 15286444

[50] Eguíluz Gracia I Martínez González de Lema B Rubio-Pérez M Ruíz-Giménez L Recio Blázquez L. Allergic reaction to undeclared lupin in a chocolate. J Investig Allergol Clin Immunol. (2015) 25:140–42. 25997311

[51] Rojas-Hijazo B Garcés MM Caballero ML Alloza P Moneo I. Unsuspected lupin allergens hidden in food. Int Arch Allergy Immunol. (2006) 141:47–50. 10.1159/000094181 16804325

[52] Reis AM Fernandes NP Marques SL Paes MJ Sousa S Carvalho F. Lupine sensitisation in a population of 1,160 subjects. Allergol Immunopathol. (2007) 35:162–63. 10.1016/S0301-0546(07)70259-8 17663927

[53] Hieta N Hasan T Mäkinen-Kiljunen S Lammintausta K. Lupin allergy and lupin sensitization among patients with suspected food allergy. Ann Allergy Asthma Immunol. (2009) 103:233–37. 10.1016/S1081-1206(10)60187-1 19788021

[54] Peeters KA Koppelman SJ Penninks AH Lebens A Bruijnzeel-Koomen CA Hefle SL. Clinical relevance of sensitization to lupine in peanut-sensitized adults. Allergy. (2009) 64:549–55. 10.1111/j.1398-9995.2008.01818.x 19076544

[55] Mennini M Dahdah L Mazzina O Fiocchi A. Lupin and other potentially cross-reactive allergens in peanut allergy. Curr Allergy Asthma Rep. (2016) 16:84. 10.1007/s11882-016-0668-8 27873194

[56] Goggin DE Mir G Smith WB Stuckey M Smith PM. Proteomic analysis of lupin seed proteins to identify conglutin Beta as an allergen, Lup an 1. J Agric Food Chem. (2008) 56:6370–77. 10.1021/jf800840u 18620408

[57] Ballabio C Peñas E Uberti F Fiocchi A Duranti M Magni C. Characterization of the sensitization profile to lupin in peanut-allergic children and assessment of cross-reactivity risk. Pediatr Allergy Immunol. (2013) 24:270–75. 10.1111/pai.12054 23551124

[58] Dooper MM Plassen C Holden L Lindvik H Faeste CK. Immunoglobulin E cross-reactivity between lupine conglutins and peanut allergens in serum of lupine-allergic individuals. J Investig Allergol Clin Immunol. (2009) 19:283–91. 19639724

[59] Alvarez-Alvarez J Guillamón E Crespo JF Cuadrado C Burbano C Rodríguez J Effects of extrusion, boiling, autoclaving, and microwave heating on lupine allergenicity. J Agric Food Chem. (2005) 53:1294–98 10.1021/jf0490145 15713055

[60] Gultekin F Doguc DK. Allergic and immunologic reactions to food additives. Clin Rev Allergy Immunol. (2013) 45:6–29. 10.1007/s12016-012-8300-8 22278172

[61] Papanikolaou I Stenger R Bessot JC de Blay F Pauli G. Anaphylactic shock to guar gum (food additive E412) contained in a meal substitute. Allergy. (2007) 62:822. 10.1111/j.1398-9995.2007.01369.x 17573733

[62] Baker G Saf S Tsuang A Nowak-Wegrzyn A. Hidden allergens in food allergy. Ann Allergy Asthma Immunol. (2018) 121:285–92. 10.1016/j.anai.2018.05.011 30219174

[63] Caponetto P Fischer J Biedermann T. Gelatin-containing sweets can elicit anaphylaxis in a patient with sensitization to galactose-α-1,3-galactose. J Allergy Clin Immunol Pract. (2013) 1:302–3. 10.1016/j.jaip.2013.01.007 24565491

[64] Wüthrich B Kägi MK Stücker W. Anaphylactic reactions to ingested carmine (E120). Allergy. (1997) 52:1133–37. 10.1111/j.1398-9995.1997.tb00189.x 9404569

[65] Takeo N Nakamura M Nakayama S Okamoto O Sugimoto N Sugiura S. Cochineal dye-induced immediate allergy: review of Japanese cases and proposed new diagnostic chart. Allergol Int. (2018) 67:496–505. 10.1016/j.alit.2018.02.012 29705083

[66] Yang WH Purchase EC. Adverse reactions to sulfites. CMAJ. (1985) 133:865–67. 4052897 PMC1346296

[67] Food and drug administration new sulfite regulations FDA Drug Bull. (1986) 16:17–18. 3817345

[68] Vally H Misso NL. Adverse reactions to the sulphite additives. Gastroenterol Hepatol Bed Bench. (2012) 5:16–23. 24834193 PMC4017440

[69] Skypala IJ Williams M Reeves L Meyer R Venter C. Sensitivity to food additives, vaso-active amines and salicylates: a review of the evidence. Clin Transl Allergy. (2015) 5:34. 10.1186/s13601-015-0078-3 26468368 PMC4604636

[70] Van Gils T Nijeboer P IJssennagger CE Sanders DS Mulder CJ Bouma G. Prevalence and characterization of self-reported gluten sensitivity in the Netherlands. Nutrients. (2016) 8:E714. 10.3390/nu8110714 27834802 PMC5133100

[71] Golley S Corsini N Topping D Morell M Mohr P. Motivations for avoiding wheat consumption in Australia: results from a population survey. Public Health Nutr. (2015) 18:490–99. 10.1017/S1368980014000652 24739252 PMC10271735

[72] Prados-Castaño M Piñero-Saavedra M Leguisamo-Milla S Pastor C Cuesta P Bartolomé B. Anaphylaxis due to oat ingestion. J Investig Allergol Clin Immunol. (2016) 26:68–9. 27012025

[73] Boussault P Léauté-Labrèze C Saubusse E Maurice-Tison S Perromat M Roul S. Oat sensitization in children with atopic dermatitis: prevalence, risks and associated factors. Allergy. (2007) 62:1251–56. 10.1111/j.1398-9995.2007.01527.x 17919139

[74] Hemmer W Sesztak-Greinecker G Wöhrl S Wantke F. Food allergy to millet and cross-reactivity with rice, corn and other cereals. Allergol Int. (2017) 66:490–92. 10.1016/j.alit.2016.11.002 27939330

[75] Lee SY Ahn K Kim J Jang GC Min TK Yang HJ. A multicenter retrospective case study of anaphylaxis triggers by age in Korean children. Allergy Asthma Immunol Res. (2016) 8:535–40. 10.4168/aair.2016.8.6.535 27582405 PMC5011054

[76] Park K Jeong K Lee S. Clinical and laboratory findings of childhood buckwheat allergy in a single tertiary hospital. Korean J Pediatr. (2016) 59:402–7. 10.3345/kjp.2016.59.10.402 27826326 PMC5099287

[77] Badiu I Olivieri E Montagni M Guida G Mietta S Pizzimenti S. Italian study on buckwheat allergy: prevalence and clinical features of buckwheat-sensitized patients in Italy. Int J Immunopathol Pharmacol. (2013) 26:801–6. 10.1177/039463201302600328 24067481

[78] Sammut D Dennison P Venter C Kurukulaaratchy RJ. Buckwheat allergy: a potential problem in 21st century Britain. BMJ Case Rep. (2011) 2011:bcr0920114882. 10.1136/bcr.09.2011.4882 22674117 PMC3214221

[79] Kasera R Niphadkar PV Saran A Mathur C Singh AB. First case report of anaphylaxis caused by Rajgira seed flour (*Amaranthus paniculatus*) from India: a clinico-immunologic evaluation. Asian Pac J Allergy Immunol. (2013) 31:79–83. 23517398

[80] Astier C Moneret-Vautrin DA Puillandre E Bihain BE. First case report of anaphylaxis to quinoa, a novel food in France. Allergy. (2009) 64:819–20. 10.1111/j.1398-9995.2009.01980.x 19220209

[81] Hong J Convers K Reeves N Temprano J. Anaphylaxis to quinoa. Ann Allergy Asthma Immunol. (2013) 110:60–1. 10.1016/j.anai.2012.10.016 23244665

[82] Micozzi S Infante S Fuentes-Aparicio V Álvarez-Perea A Zapatero L. Celiac disease and wheat allergy: a growing association? Int Arch Allergy Immunol. (2018) 176:280–82. 10.1159/000489305 29847816

[83] Martín-Muñoz MF Rivero D Díaz Perales A Polanco I Quirce S. Wheat allergy in celiac children. Pediatr Allergy Immunol. (2016) 27:102–5. 10.1111/pai.12487 26360318

[84] Berneder M Bublin M Hoffmann-Sommergruber K Hawranek T Lang R. Allergen chip diagnosis for soy-allergic patients: Gly m 4 as a marker for severe food-allergic reactions to soy. Int Arch Allergy Immunol. (2013) 161:229–33. 10.1159/000345970 23548307 PMC4739502

[85] Jacobson MF DePorter J. Self-reported adverse reactions associated with mycoprotein (Quorn-brand) containing foods. Ann Allergy Asthma Immunol. (2018) 120:626–30. 10.1016/j.anai.2018.03.020 29567357

[86] Hoff M Trüeb RM Ballmer-Weber BK Vieths S Wuethrich B. Immediate-type hypersensitivity reaction to ingestion of mycoprotein (Quorn) in a patient allergic to molds caused by acidic ribosomal protein P2. J Allergy Clin Immunol. (2003) 111:1106–10. 10.1067/mai.2003.1339 12743577

[87] Katona SJ Kaminski ER. Sensitivity to Quorn mycoprotein (*Fusarium venenatum*) in a mould allergic patient. J Clin Pathol. (2002) 55:876–77. 10.1136/jcp.55.11.876-a 12401831 PMC1769805

[88] Acker WW Plasek JM Blumenthal KG Lai KH HTopaz M Seger DL. Prevalence of food allergies and intolerances documented in electronic health records. J Allergy Clin Immunol. (2017) 140:1587–91.e1. 10.1016/j.jaci.2017.04.006 28577971 PMC7059078

[89] Skypala IJ Calderon MA Leeds AR Emery P Till SJ Durham SR. Development and validation of a structured questionnaire for the diagnosis of Oral Allergy Syndrome in subjects with seasonal allergic rhinitis during the UK birch pollen season. Clin Exp. Allergy. (2011) 41:1001–11. 10.1111/j.1365-2222.2011.03759.x 21518043

[90] Larramendi CH García-Abujeta JL Vicario S García-Endrino A López-Matas MA García-Sedeño MD. Goji berries (*Lycium barbarum*.): risk of allergic reactions in individuals with food allergy. J Investig Allergol Clin Immunol. (2012) 22:345–50. 23101309

[91] Almeida EM Bartolomé B Faria EG Sousa NG Luís AS. Pomegranate anaphylaxis due to cross-reactivity with Peach LTP (Pru p 3). Allergol Immunopathol. (2015) 43:104–6. 10.1016/j.aller.2013.07.013 24275183

[92] Gunawardana NC. Risk of anaphylaxis in complementary and alternative medicine. Curr Opin Allergy Clin Immunol. (2017) 17:332–37. 10.1097/ACI.0000000000000384 28731887

[93] Khalili B Bardana EJ Jr Yunginger JW. Psyllium-associated anaphylaxis and death: a case report and review of the literature. Ann Allergy Asthma Immunol. (2003) 91:579–84. 10.1016/S1081-1206(10)61538-4 14700444

[94] Shadick NA Liang MH Partridge AJ Bingham C Wright E Fossel AH. The natural history of exercise-induced anaphylaxis: survey results from a 10-year follow-up study. J Allergy Clin Immunol. (1999) 104:123–27. 10.1016/S0091-6749(99)70123-5 10400849

[95] Christensen MJ Eller E Mortz CG Brockow K Bindslev-Jensen C. Exercise Lowers Threshold and Increases Severity, but Wheat-Dependent, Exercise-Induced Anaphylaxis Can Be Elicited at Rest. J Allergy Clin Immunol Pract. (2018) 6:514–20. 10.1016/j.jaip.2017.12.023 29524997

[96] Romano A Scala E Rumi G Gaeta F Caruso C Alonzi C. Lipid transfer proteins: the most frequent sensitizer in Italian subjects with food-dependent exercise-induced anaphylaxis. Clin Exp Allergy. (2012) 42:1643–53. 10.1111/cea.12011 23106665

[97] Pascal M Muñoz-Cano R Reina Z Palacín A Vilella R Picado C. Lipid transfer protein syndrome: clinical pattern, cofactor effect and profile of molecular sensitization to plant-foods and pollens. Clin Exp Allergy. (2012) 42:1529–39. 10.1111/j.1365-2222.2012.04071.x 22994350

[98] Cardona V Luengo O Garriga T Labrador-Horrillo M Sala-Cunill A Izquierdo A. Co-factor-enhanced food allergy. Allergy. (2012) 67:1316–18. 10.1111/j.1398-9995.2012.02877.x 22845005

[99] Versluis A van Os-Medendorp H Blom WM Michelsen-Huisman AD Castenmiller JJM Noteborn HPJM Potential cofactors in accidental food allergic reactions are frequently present but may not influence severity and occurrence. Clin Exp Allergy. (2018) 49:207–15. 10.1111/cea.13282 30244525

[100] Asaumi T Ebisawa M. How to manage food dependent exercise induced anaphylaxis (FDEIA). Curr Opin Allergy Clin Immunol. (2018) 18:243–47. 10.1097/ACI.0000000000000442 29629955

[101] Bartra J Araujo G Muñoz-Cano R. Interaction between foods and nonsteroidal anti-inflammatory drugs and exercise in the induction of anaphylaxis. Curr Opin Allergy Clin Immunol. (2018) 18:310–16. 10.1097/ACI.0000000000000461 29889141

[102] Sánchez-Borges M Suárez Chacón R Capriles-Hulett A Caballero-Fonseca F Fernández-Caldas E. Anaphylaxis from ingestion of mites: pancake anaphylaxis. J Allergy Clin Immunol. (2013) 131:31–35. 10.1016/j.jaci.2012.09.026 23154081

[103] Sánchez-Borges M Fernandez-Caldas E. Hidden allergens and oral mite anaphylaxis: the pancake syndrome revisited. Curr Opin Allergy Clin Immunol. (2015) 15:337–43. 10.1097/ACI.0000000000000175 26110684

[104] Posthumus J Borish L. A 71-year-old man with anaphylaxis after eating grits. Allergy Asthma Proc. (2012) 33:110–13. 10.2500/aap.2012.33.3476 22370536 PMC3894600

[105] Canavan M Mitchell A Sharkey A Whitethorn C McNicholl B Robinson S. Oral mite anaphylaxis. QJM. (2018) 111:189–90. 10.1093/qjmed/hcx255 29309668

[106] Esquivel A Busse WW. Anaphylaxis conundrum: a trojan horse phenomenon. J Allergy Clin Immunol Pract. (2017) 5:325–29. 10.1016/j.jaip.2016.08.008 27765461 PMC5346333

[107] Pravettoni V Primavesi L Piantanida M. Anisakis simplex: current knowledge. Eur Ann Allergy Clin Immunol. (2012) 44:150–56. 23092000

[108] Heffler E Sberna ME Sichili S Intravaia R Nicolosi G Porto M. High prevalence of Anisakis simplex hypersensitivity and allergy in Sicily, Italy. Ann Allergy Asthm A Immunol. (2016) 116:146–50. 10.1016/j.anai.2015.12.014 26815707

[109] Nieuwenhuizen NE Lopata AL. Allergic reactions to Anisakis found in fish. Curr Allergy Asthma Rep. (2014) 14:455. 10.1007/s11882-014-0455-3 25039016

[110] de Gier S Verhoeckx K. Insect (food) allergy and allergens. Mol Immunol. (2018) 100:82–106. 10.1016/j.molimm.2018.03.015 29731166

[111] Tamagawa-Mineoka R Masuda K Yagami A Nakamura M Sato N Matsunaga K. Food-induced anaphylaxis in two patients who were using soap containing foodstuffs. Allergol Int. (2018) 67:427–29. 10.1016/j.alit.2018.02.013 29674155

[112] Taylor SL Baumert JL Boudreau-Romano SM. Allergic reaction from fingerprint kit attributable to unlabeled gluten, probable wheat flour. J Allergy Clin Immunol Pract. (2017) 5:479–81. 10.1016/j.jaip.2016.06.018 27452885

[113] Fritz SB Gold BL. Buckwheat pillow-induced asthma and allergic rhinitis. Ann Allergy Asthma Immunol. (2003) 90:355–58. 10.1016/S1081-1206(10)61807-8 12669902

[114] Makatsori M Scadding GW Skypala I Durham SR. Silk contact anaphylaxis. Contact Dermatitis. (2014) 71:314–5. 10.1111/cod.12289 25345796

[115] Heaps A Carter S Selwood C Moody M Unsworth J Deacock S. The utility of the ISAC allergen array in the investigation of idiopathic anaphylaxis. Clin Exp Immunol. (2014) 177:483–90. 10.1111/cei.12334 24654858 PMC4226599

[116] Skypala IJ Venter C Meyer R de Jong NW Fox AT Groetch M Allergy-focussed Diet History Task Force of the European Academy of Allergy and Clinical Immunology. The development of a standardised diet history tool to support the diagnosis of food allergy. Clin Transl Allergy. (2015) 5:7 10.1186/s13601-015-0050-2 25741437 PMC4349299

[117] Fernández-Rivas M. Fruit and vegetable allergy. Chem Immunol Allergy. (2015) 101:162–70. 10.1159/000375469 26022876

[118] Martelli A De Chiara A Corvo M Restani P Fiocchi A. Beef allergy in children with cow's milk allergy; cow's milk allergy in children with beef allergy. Ann Allergy Asthma Immunol. (2002) 89:38–43. 10.1016/S1081-1206(10)62121-7 12487203

[119] de Silva R Dasanayake WMDK Wickramasinhe GD Karunatilake C Weerasinghe N Gunasekera P. Sensitization to bovine serum albumin as a possible cause of allergic reactions to vaccines. Vaccine. (2017) 35:1494–500. 10.1016/j.vaccine.2017.02.009 28216185

[120] Steinke JW Platts-Mills TA Commins SP. The alpha-gal story: lessons learned from connecting the dots. J Allergy Clin Immunol. (2015) 135:589–96. 10.1016/j.jaci.2014.12.1947 25747720 PMC4600073

[121] Commins SP. Invited commentary: alpha-gal allergy: tip of the iceberg to a pivotal immune response. Curr Allergy Asthma Rep. (2016) 16:61. 10.1007/s11882-016-0641-6 27520937

[122] Jappe U Vieths S. Lupine, a source of new as well as hidden food allergens. Mol Nutr Food Res. (2010) 54:113–26. 10.1002/mnfr.200900365 20013885

